# SIAH1-mediated RPS3 ubiquitination contributes to chemosensitivity in epithelial ovarian cancer

**DOI:** 10.18632/aging.204211

**Published:** 2022-08-08

**Authors:** Lu Chen, Wujiang Gao, Chunli Sha, Meiling Yang, Li Lin, Taoqiong Li, Hong Wei, Qi Chen, Jie Xing, Mengxue Zhang, Shijie Zhao, Wenlin Xu, Yuefeng Li, Xiaolan Zhu

**Affiliations:** 1 Reproductive Medicine Center, The Fourth Affiliated Hospital of Jiangsu University, Zhenjiang, Jiangsu, China; 2 Department of Central laboratory, The Fourth Affiliated Hospital of Jiangsu University, Zhenjiang, Jiangsu, China; 3 Department of Radiology, Affiliated Hospital of Jiangsu University, Zhenjiang, Jiangsu, China; 4 International Genome Center of Jiangsu University, Zhenjiang, Jiangsu, China; 5 Obstetrics and Gynecology, The First People’s Hospital of Nantong City, Nantong, Jiangsu, China

**Keywords:** EOC, chemoresistance, SIAH1, RPS3, NF-κB

## Abstract

The E3 ligase SIAH1 is deregulated in human cancers and correlated with poor prognosis, but its contributions to chemoresistance in epithelial ovarian cancer (EOC) are not evident. Herein we found that SIAH1 was decreased in EOC tumour tissues and cell lines and negatively correlated with the RPS3 levels. SIAH1 overexpression suppressed tumour cell growth, colony formation, invasion, metastasis, and cisplatin resistance *in vivo* and *in vitro*. SIAH1 promoted RPS3 ubiquitination and degradation using the RING-finger domain, and these steps were required for RPS3 localization to the cytoplasm, which led to subsequent NF-κB inactivation and thereby conferred chemosensitivity. Moreover, ectopic expression of RPS3 or depletion of RPS3 ubiquitination mediated by SIAH1 via the K214R mutant significantly impaired cisplatin-induced tumour suppression in cells stably expressing SIAH1. Together, our findings reveal a tumour suppressor function of SIAH1 and provide evidence showing that the SIAH1-RPS3-NF-κB axis may act as an appealing strategy for tackling treatment resistance in EOC.

## INTRODUCTION

Mainly due to the development of chemoresistance, most epithelial ovarian cancers (EOCs) are prone to treatment failure despite all current standard treatments [1], and these cancers are associated with the highest risk of death among gynaecological malignancies [2, 3]. Hence, understanding the regulatory mechanism of chemoresistance, which is the bottleneck of EOC treatment, is critical [4].

As a crucial player, the ubiquitin-proteasome pathway manages the steady-state protein levels to supervise multifarious biological processes, including the cell cycle, cellular proliferation, apoptosis, and DNA damage response, which are linked with oncogenesis, cancer development, drug resistance and prognosis [5, 6]. In the ubiquitination cascade, E3 ligases execute the terminal step and determine which proteins become ubiquitylated with the help of specifically binding the substrate protein. Due to their capacity to regulate protein stability and functions, E3 ligases are considered promising drug targets [7]. As a member of the E3 ubiquitin ligase family, seven in absentia homologue 1 (SIAH1) contains two zinc-finger domains, a RING-finger domain, and a substrate-binding domain. Of these domains, the RING-finger domain is critical to ubiquitin ligase activity [8]. SIAH1 reportedly behaves as a tumour suppressor during tumourigenesis by binding to numerous proteins, such as NcoR, TRAF, β-catenin, c-Myb, APC, and Kid, and thereby triggers their ubiquitylation and degradation via the ubiquitin–proteasome pathway [9–12]. In addition, SIAH1 has been implicated in cancer-related signalling pathways, including cell apoptosis (β-catenin, Sec6) [13, 14], DNA damage repair (c-Abl, HIPK2, and TRF2) [15–17] and the *in vivo* hypoxia response (PHD3, HIF-1a, and IL-17) [13, 18, 19]. Thus, SIAH1 has been proposed as a promising therapeutic target in cancer treatment [20].

As one of the eukaryotic ribosome 40S subunits, ribosomal protein S3 (RPS3) is critical for regulating the maturation of ribosomes and the initiation of translation with the eukaryotic initiation factors elF2 and elF3 [21, 22]. Independent of the activities of ribosomes, RPS3 has a variety of extraribosomal functions, such as DNA repair [23–28], cell signalling [29–32], apoptosis/survival [33], host-pathogen interactions [34, 35] and transcriptional regulation [36–38]. As previously reported, RPS3 interacts with the p65 subunit of nuclear factor kappa B (NF-κB) through its K homology domain, which results in NF-κB-induced transcriptional activation [38–40]. NF-κB adjusts the expression of genes (PTOV1, c-Myb, TRIM52) [41–43] related to numerous processes that play pivotal roles in the development and progression of cancer, such as proliferation, migration and apoptosis [44]. Many findings have indicated that NF-κB pathway activation promotes ovarian cancer chemotherapy resistance [41–43].

Above all, this project aimed to explore the role of SIAH1 in EOC drug resistance, identify its interacting molecular chaperones, and regulate the NF-κB pathway, with the objective of identifying new targets for EOC drug resistance reversal and prevention.

## RESULTS

### SIAH1 deregulation is associated with drug resistance in EOC

**Figure 1:**
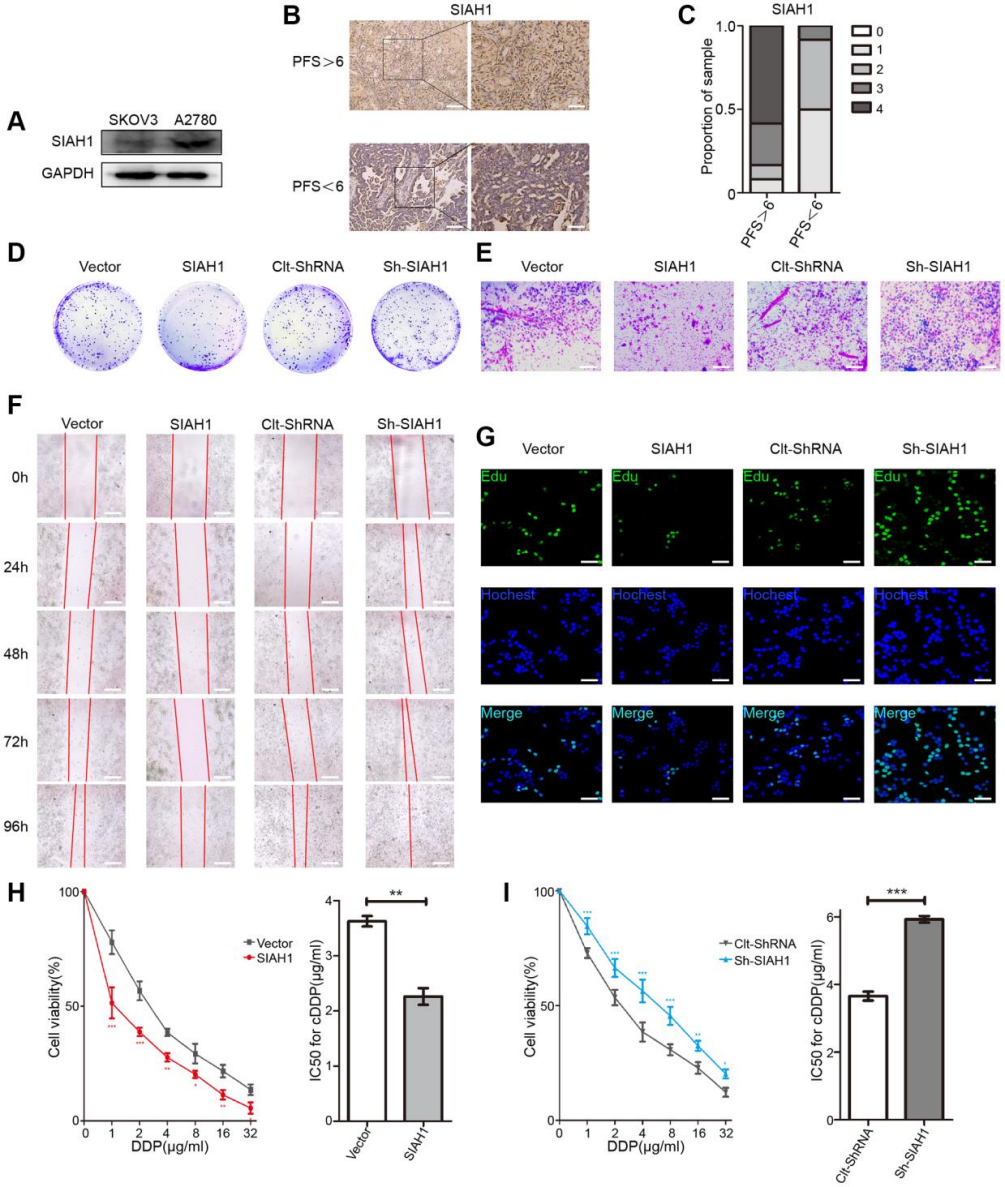
**SIAH1 sensitizes ovarian cancer cells to cDDP.** (**A**) Western blotting for SIAH1 in SKOV3 and A2780 cells. (**B**) Representative images of immunohistochemical staining for SIAH1 in tumour specimens from ovarian cancer patients with PFS > 6 months vs. PFS < 6 months. Scale bar: 200 μm (left) and 100 μm (right). (**C**) Staining was assessed and scored on a scale of 0 (<5% staining) to 4 (>75% staining). The quantification of IHC staining (*n* = 24; PFS > 6, *n* = 12; PFS < 6, *n* = 12) was shown. The results from a Cell Colony formation assay (**D**) and Transwell assay (**E**) of A2780 cells transfected with Vector, SIAH1, Clt-shRNA and sh-SIAH1 were shown. Scale bar: 400 μm. A wound-healing assay was used to assess the effects of SIAH1 on cellular motility over time as shown (0, 24, 48, 72 and 96 h; Scale bar: 400 μm) (**F**). The results from a Cell Edu assay (Scale bar: 200 μm) (**G**), Cell Viability (**H**, **I** left panels) and IC50 for cDDP (**H**, **I** right panels) in A2780 cells were shown.^*^p < 0.05, ^**^p < 0.01, ^***^p < 0.001.

To determine the effect of SIAH1 on the development and progression of EOC, we explored the expression of SIAH1 in cDDP-sensitive EOC cells (A2780) and cDDP-resistant cells (SKOV3). The results showed that A2780 cells exhibited strong expression of SIAH1, whereas SKOV3 cells lacked SIAH1 expression (Figure 1A), which demonstrated that SIAH1 may be associated with drug resistance in EOC. In addition, the SIAH1 protein levels were clearly increased in tumours obtained from patients with progression-free survival (PFS) > 6 months (clinically described as cDDP sensitive), whereas its expression was reduced in tumours obtained from patients with PFS < 6 months (cDDP resistant; Figure 1B, 1C and Supplementary Figure 1B). This finding further suggested that SIAH1 may play a functional role in EOC.

To further clarify the role of SIAH1 in the progression of EOC, we transfected EOC cells with SIAH1, Vector, sh-SIAH1 and Clt-shRNA respectively (Supplementary Figure 1A), and found that SIAH1 overexpression decreased the quantity of cell colonies and cell viability, impaired cell migration and invasion, increased the apoptosis rate and declined the chemoresistance of EOC cells. In contrast, SIAH1 knockdown showed a greater ability to increase cell proliferation and cell viability, strengthen cell migration and invasion, inhibit cell apoptosis and enhance the drug resistance of EOC cells (Figure 1D–1I and Supplementary Figure 1C–1F). These results indicate that SIAH1 renders EOC cells sensitive to cDDP.

### Identification of RPS3 as an interaction partner of SIAH1

**Figure 2:**
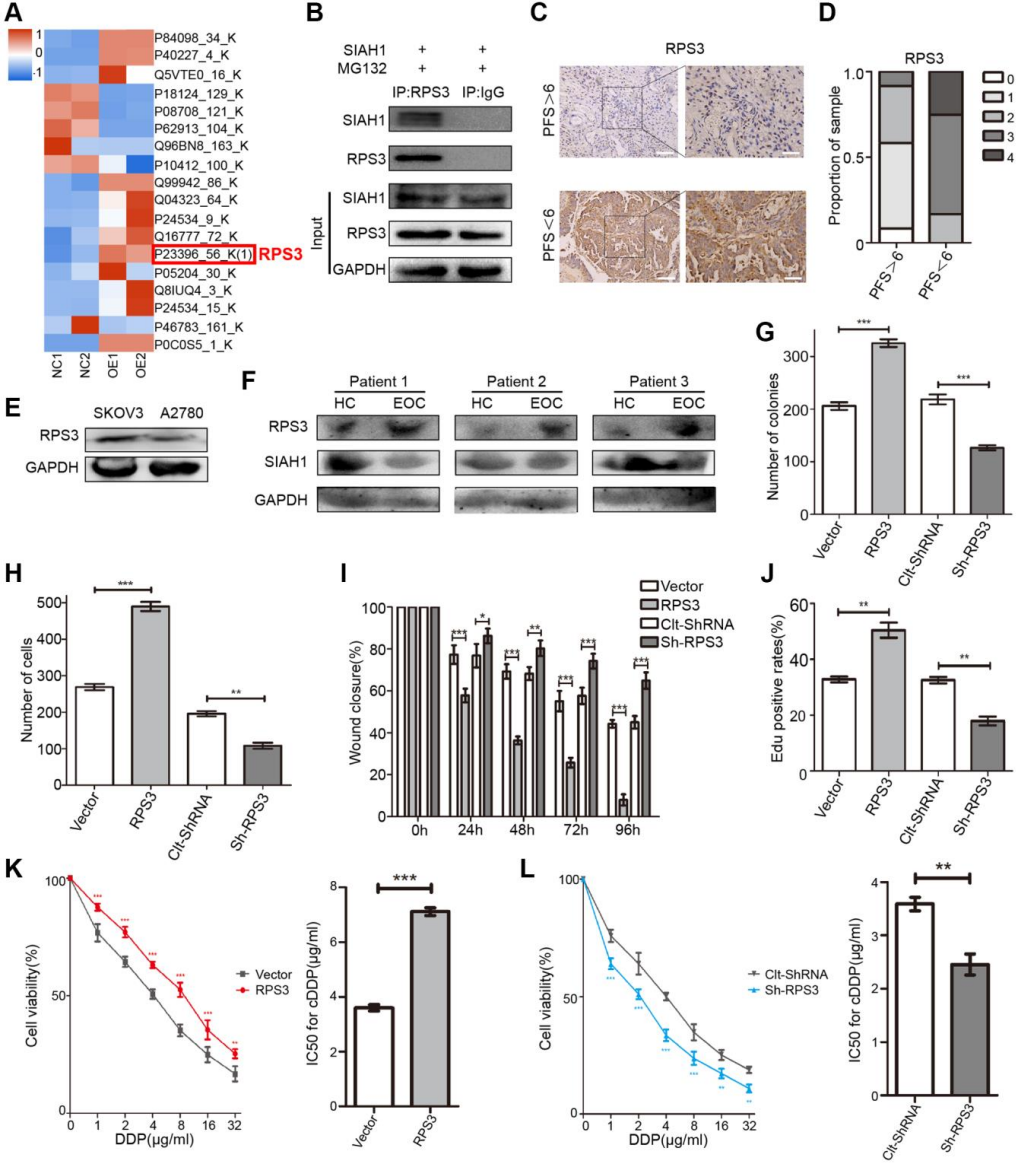
**Identification of RPS3 as an interaction partner of SIAH1.** (**A**) The heatmap showed that the protein level of RPS3 was down-regulated after SIAH1 overexpression in EOC cells. (**B**) Pull down of the RPS3 protein with SIAH1 antibody and IgG antibody in A2780 cells transfected with SIAH1. Cells were treated with 10 μM MG132 for 6 h before cell lysis. (**C**) Representative images of immunohistochemical staining for RPS3 in tumour specimens from ovarian cancer patients with PFS > 6 months vs. PFS < 6 months. Scale bar: 200 μm (left) and 100 μm (right). (**D**) The staining was assessed and scored on a scale of 0 (<5% staining) to 4 (>75% staining). The quantification of IHC staining (*n* = 24; PFS > 6, *n* = 12; PFS < 6, *n* = 12) was shown. (**E**) Protein level of RPS3 in SKOV3 and A2780 cells. (**F**) Protein levels of SIAH1 and RPS3 in serum samples from healthy controls (HC) and EOC patients (EOC). A2780 cells were separately transfected with Vector, RPS3, Clt-shRNA and sh-RPS3 for 48 h, the number of Cell Colonies was determined (**G**), the cell number from the Transwell assay was obtained (**H**), the Wound closure percentage was calculated (0, 24, 48, 72 and 96 h) (**I**), and the Edu positive rates (**J**), Cell Viability (**K**, **L** left panels), IC50 for cDDP (**K**, **L** right panels) were obtained. ^*^p < 0.05, ^**^p < 0.01, ^***^p < 0.001.

To gain prime insight into SIAH1 expression patterns, we identified potential SIAH1-binding proteins in HEK-293T cells transfected with SIAH1, Vector, sh-SIAH1 and Clt-shRNA plasmids through co-immunoprecipitation assays using the anti-SIAH1 antibody. The total immunoprecipitates were then subjected to Liquid Chromatography-Mass Spectrometry/Mass Spectrometry (LC-MS/MS) (Figure 2A and Supplementary Figure 2A–2G). The potential SIAH1-interacting proteins were selected based on the following 2 criteria: (1) more than 2 unique protein peptides, and (2) sequence coverage of proteins greater than 70%. Consequently, 20 potential SIAH1-interacting proteins were identified (Supplementary Table 3), and interestingly, approximately one-fourth (5 from 20) of these proteins belong to the ribosomal protein family. Additionally, it has been reported that RPS3 is essential for the induction of chemoresistance in cancer cells [45, 46]. Therefore, we selected RPS3 as our research object. Through Co-IP assays, an interaction between SIAH1 and RPS3 was confirmed in HEK-293T cells transfected with SIAH1, and the immunoprecipitates pulled down by anti-SIAH1 antibody were detected (Figure 2B). As a control, no RPS3 protein was detected in the immunoprecipitates that were pulled down with anti-IgG antibody.

### RPS3 confers chemoresistance in EOC

To clarify the role of RPS3 in EOC drug resistance, RPS3 expression was assessed in primary EOC specimens originating from 24 EOC patients. cDDP-resistant patients (PFS < 6) showed higher RPS3 expression, and cDDP-sensitive patients (PFS > 6) exhibited lower RPS3 expression (Figure 2C, 2D and Supplementary Figure 3B). Analogous to the results found for high SIAH1 expression, low RPS3 expression was associated with longer PFS. Additionally, we observed high RPS3 expression in cDDP-resistant EOC cells (Figure 2E). To further confirm the clinical relevance of SIAH1 and RPS3, we assessed the expression of SIAH1 and RPS3 protein in serum obtained from 12 subjects, including 6 idiopathic cases of EOC patients (EOCs) and 6 age-matched healthy female controls (HCs). The results showed higher SIAH1 expression in HCs and higher RPS3 expression in EOCs (Figure 2F). After the promotion or inhibition of RPS3 expression by transfection with RPS3-specific plasmids (Supplementary Figure 3A), the short-term impact of RPS3 on cell survival was evaluated through a cell viability assay (CCK-8 assay). RPS3 overexpression significantly enhanced cDDP resistance in EOC cells, which suggested that RPS3 confers chemoresistance in EOC cells (Figure 2K, 2L). To evaluate the long-term impact of RPS3 on cell proliferation after treatment with cDDP, a colony formation assay was performed. The number of colonies in the presence of cDDP was significantly increased by RPS3 overexpression and was greatly decreased by RPS3 knockdown (Figure 2G and Supplementary Figure 3C). Furthermore, the overexpression of RPS3 inhibited cell apoptosis, strengthened cell migration and invasion, and enhanced cDDP resistance (Figure 2H–2J and Supplementary Figure 3D–3F).

### SIAH1 sensitizes EOC cells to cDDP through the regulation of RPS3

**Figure 3:**
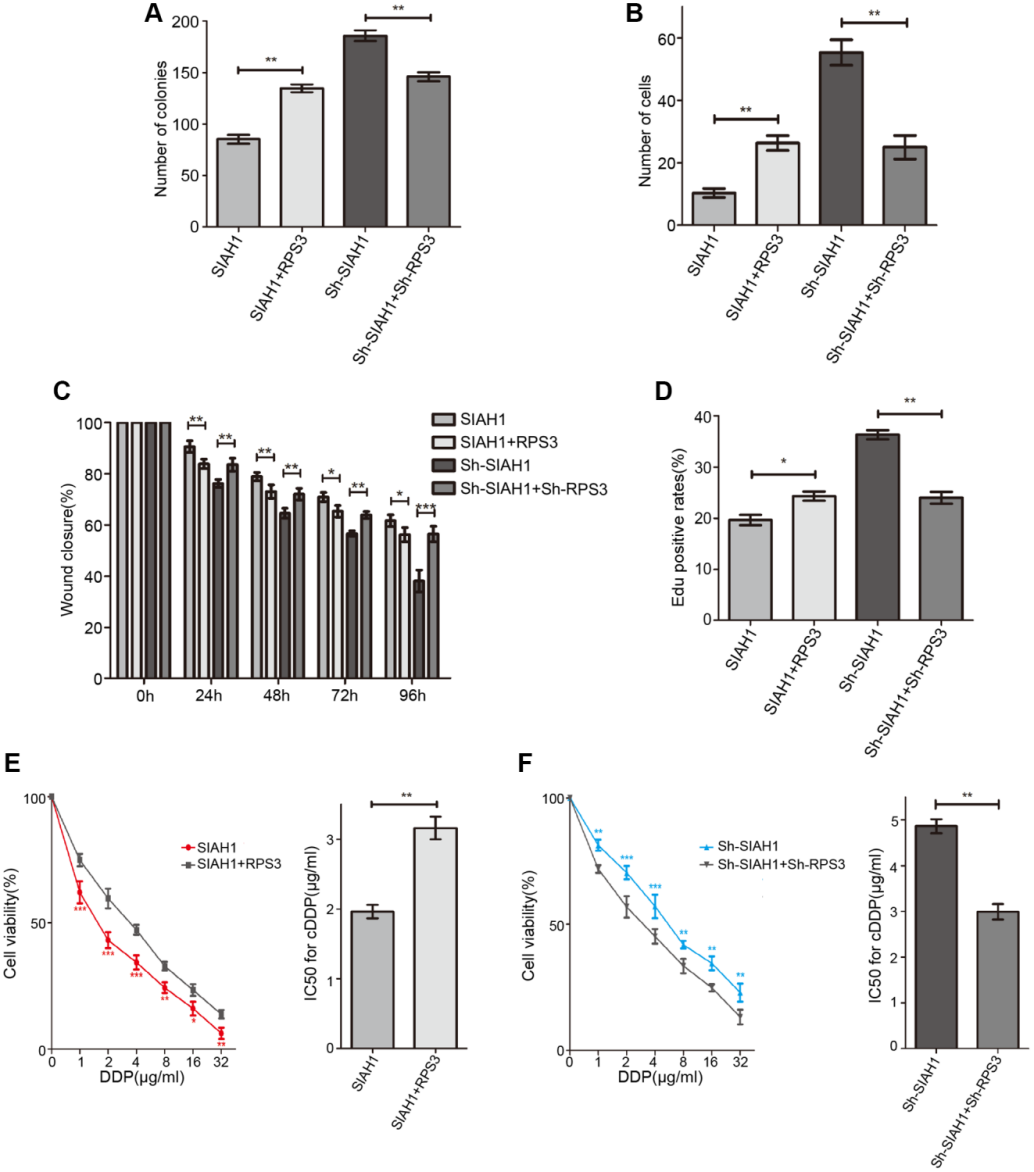
**SIAH1 down-regulates the protein level of RPS3.** A2780 cells were separately transfected with SIAH1, RPS3, sh-SIAH1 or sh-RPS3 for 48 h, the number of Cell Colonies was determined (**A**), the cell number from the Transwell assay was obtained (**B**), the Wound closure percentage was calculated (0, 24, 48, 72 and 96 h) (**C**), and the Edu positive rates (**D**), cell viability (**E**, **F** left panels), and IC50 for cDDP (**E**, **F** right panels) were measured. ^*^p < 0.05, ^**^p < 0.01, ^***^p < 0.001.

We then sought to further confirm that SIAH1-mediated EOC chemotherapy sensitization is related to its partner, RPS3. EOC cells were transfected with SIAH1, SIAH1+RPS3, sh-SIAH1 and sh-SIAH1+sh-RPS3 plasmids. SIAH1-expressing EOC cells exhibited reduced cell viability, impairments in cell migration and invasion, a higher apoptosis rate and decreased chemoresistance, and these effects were mitigated by RPS3 transfection. In contrast, the increases in cell proliferation and cell viability, the reinforcement of cell migration and invasion, the inhibition of cell apoptosis and the enhancement of chemoresistance in EOC cells transfected with sh-SIAH1 were repaired by sh-RPS3 (Figure 3A–3F). These results indicate that the SIAH1-induced sensitivity of EOC cells to cDDP was attenuated by RPS3.

### SIAH1 facilitates RPS3 degradation via the ubiquitin-proteasome pathway

**Figure 4:**
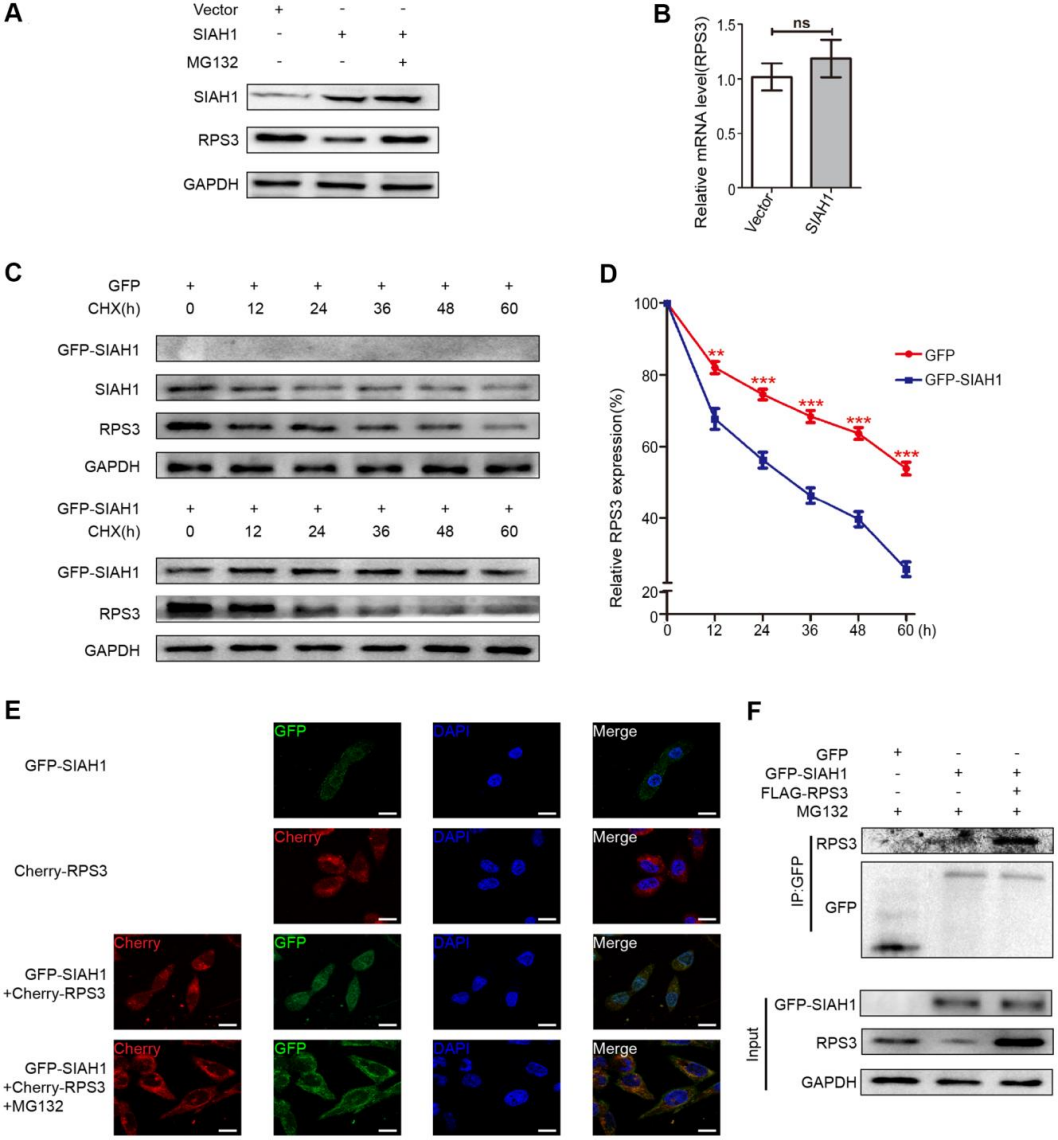
**SIAH1 induces degradation of RPS3 protein.** (**A**) A2780 cells were transfected with the Vector, SIAH1. Cells were treated with or without 10 μM MG132 for 6 h before cell lysis. and the resulting cell lysates were subjected to Western blotting. (**B**) RPS3 mRNA levels was detected in A2780 cells with SIAH1 overexpression. (**C**, **D**) The RPS3 protein half-life was assayed by using CHX (30 μg/ml) in HEK293T cells transfected with GFP or GFP-SIAH1 plasmid. The relative remaining RPS3 protein levels following CHX treatment at each time point were calculated accordingly. (**E**) Colocalization of RPS3 and SIAH1. The GFP-SIAH1 or Cherry-RPS3 plasmids were transfected into A2780 cells. Cells were treated with or without 10 μM MG132 for 6 h before cell lysis. GFP-SIAH1 was detected using a fluorescence microscope with an excitation wavelength of 488 nm. Cherry-RPS3 was detected with an excitation wavelength of 556 nm. The cell nuclei were stained with DAPI. Scale bar: 50 μm. (**F**) A2780 cells were transfected with plasmids as indicated, and the RPS3 protein was pull down with GFP antibody. Cell lysates were subjected to Western blotting. ^*^p < 0.05, ^***^p < 0.001.

To detect whether RPS3, which was reduced by SIAH1, is involved in proteasome-dependent degradation, A2780 cells transfected with SIAH1 plasmid were treated with MG132 (proteasome inhibitor), and the expression levels of RPS3 in SIAH1-expressing cells were detected. The results showed that the SIAH1-mediated down-regulation of RPS3 protein expression was attenuated by MG132 treatment (Figure 4A), and SIAH1 had no effect on RPS3 gene expression (Figure 4B). Furthermore, the half-life of RPS3 was decreased by SIAH1 in EOC cells treated with CHX (Figure 4C, 4D). These above results indicated that SIAH1 destabilizes RPS3 proteins through the ubiquitin-proteasome pathway in EOC cells. Consistently, the co-localization of SIAH1 and RPS3 in EOC cells was discovered by confocal microscopy (Figure 4E). Moreover, the results shown in Figure 4F indicated that SIAH1 interacted with both endogenous and exogenous RPS3, which further supports the above-described conclusion.

**Figure 5:**
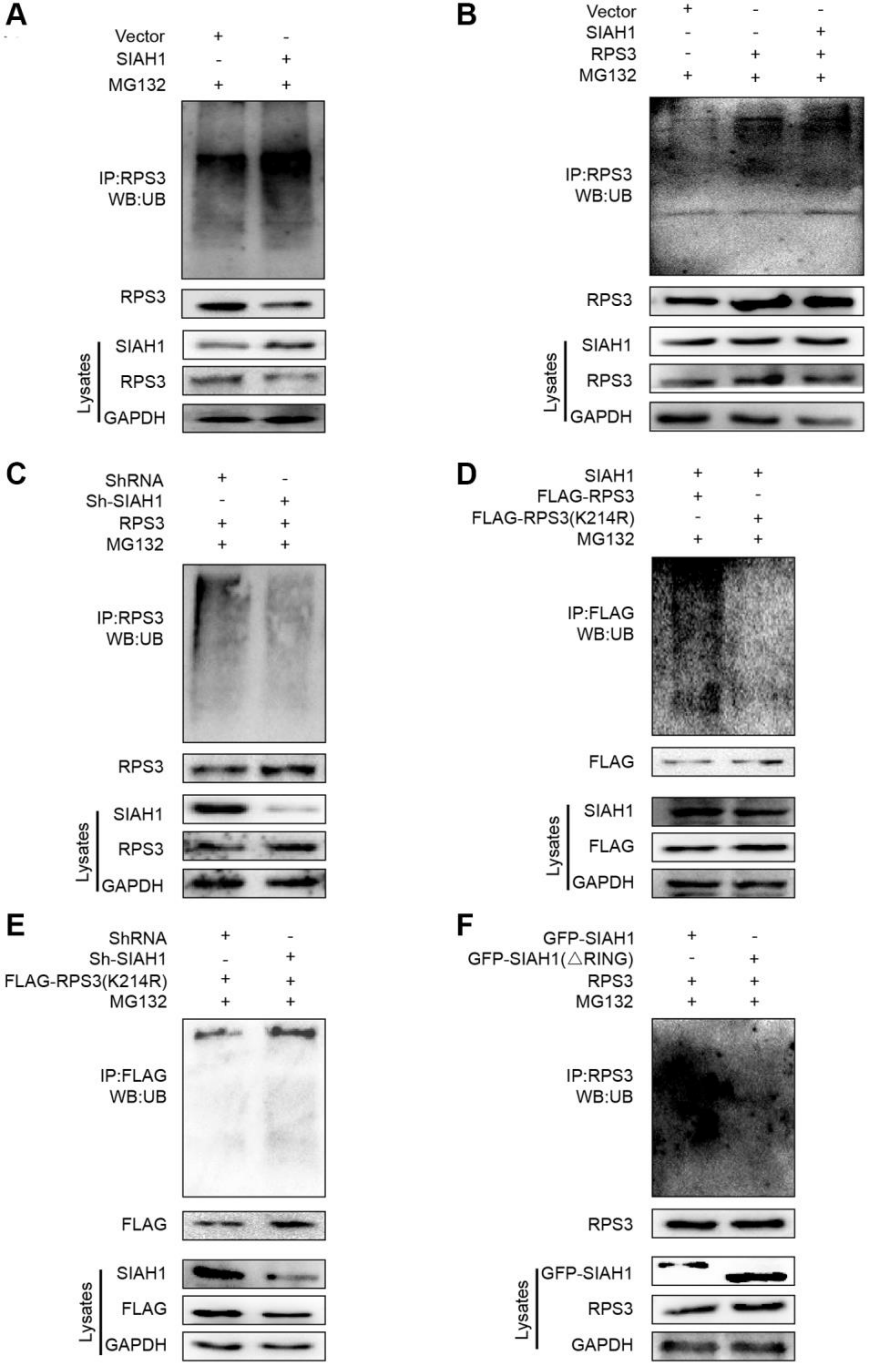
**SIAH1 is an E3 ligase to induce RPS3 ubiquitination.** (**A**–**F**) HEK293T cells were transfected plasmids as indicated for 36 h. Cell lysates were subjected to denatured immunoprecipitation and Western blotting. Cells were treated with MG132 (10 μM) for 6 h before cell lysis. (**A**) SIAH1 overexpression induced ubiquitination and degradation of endogenous RPS3. (**B**) SIAH1 overexpression induced ubiquitination and degradation of exogenous RPS3. (**C**) When SIAH1 was knocked down, the ubiquitination level of RPS3 was significantly reduced. (**D**) Upon SIAH1 expression, FLAG-RPS3 (K214R) had a significantly lower ubiquitination than the wild type (FLAG-RPS3). (**E**) SIAH1 KD, induced no significant decrease in the ubiquitination level of FLAG-RPS3 (K214R). (**F**) In SIAH1-inhibited A2780 cells, mutation of the RING finger domain of SIAH1 significantly reduced the ubiquitination level of RPS3.

To confirm whether SIAH1 can induce RPS3 ubiquitination, we conducted immunoprecipitation and Western blotting experiments. The outcomes showed that SIAH1 markedly induced endogenous and exogenous RPS3 ubiquitination, which was revealed by immunoprecipitation analysis (Figure 5A, 5B). In contrast, the knockdown of SIAH1 resulted in inhibition of RPS3 ubiquitination (Figure 5C).

### SIAH1 ubiquitinates RPS3 at its K214

To identify the potential chief ubiquitin site of RPS3, we re-analysed the LC-MS/MS results and found 11 potential ubiquitination sites (Supplementary Table 4). Based on their scores, *P* values and ubiquitination levels in the context of SIAH1 overexpression or knockdown, K214 was selected as the best site. We then mutated the lysine residue (K214) to arginine and measured the resulting ubiquitination levels, which verified the ubiquitin modification of RPS3 at K214 (Figure 5D). Additionally, ubiquitin did not decrease after SIAH1 knockdown (Figure 5E). The above mentioned data suggest that SIAH1 ubiquitinates RPS3 at K214 in a proteasome-dependent manner.

### The RING finger domain of SIAH1 is required for RPS3 ubiquitination

To determine the key domain of SIAH1 mediating RPS3 ubiquitination, we co-transfected RPS3 with SIAH1 lacking the RING domain (GFP-SIAH1∆RING) and therefore could not achieve E3-mediated ubiquitin transfer on its targets to demonstrate the direct impact of SIAH1 E3 ligase activity. We discovered that deletion of the RING domain abolished the ability of SIAH1 to ubiquitinate RPS3 (Figure 5F). These results imply that the RING finger domain of SIAH1 is needed for RPS3 ubiquitination and are consistent with the notion that SIAH1 acts as a RING finger E3 ubiquitin ligase.

### SIAH1 inhibits the NF-κB pathway via RPS3 downregulation

**Figure 6:**
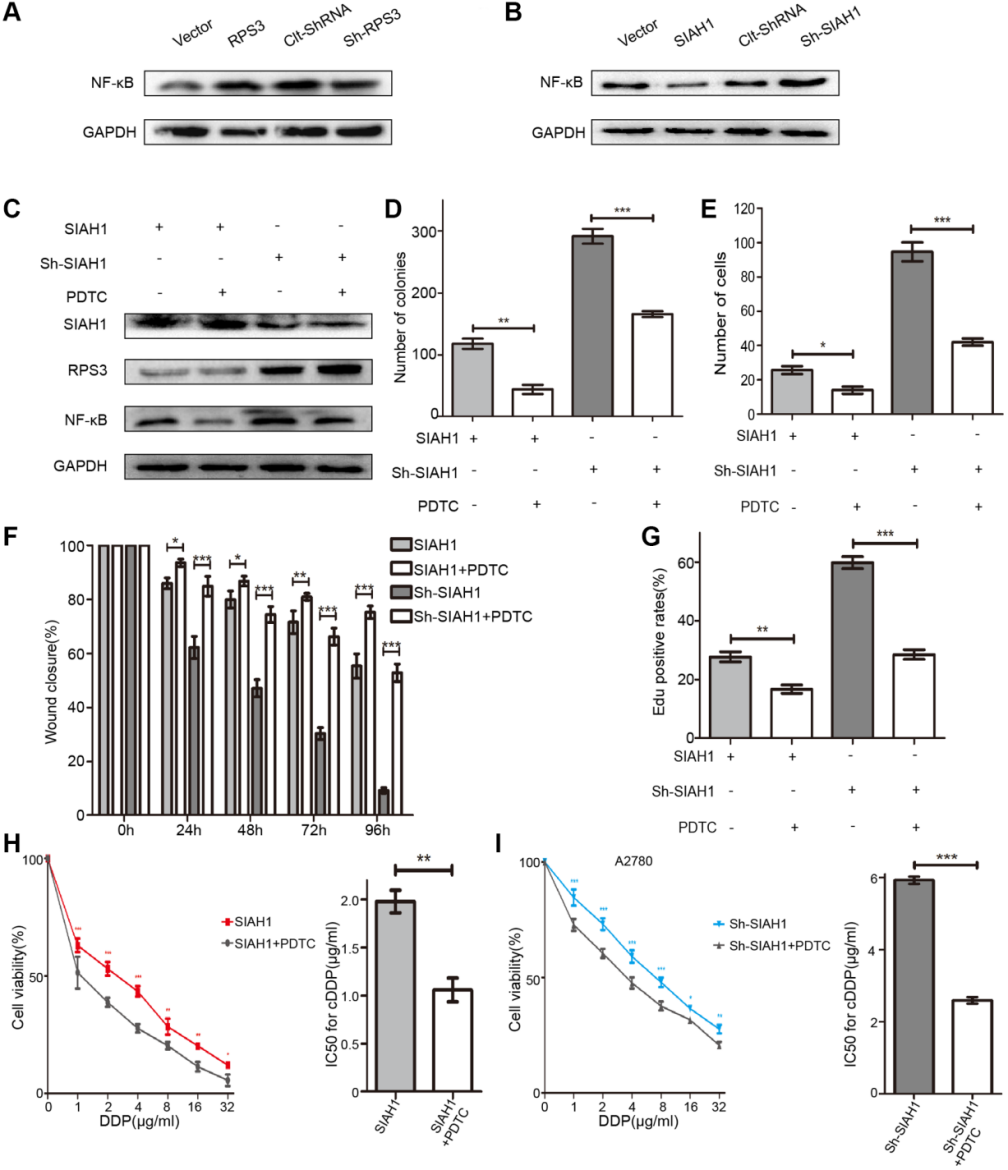
**SIAH1 inhibits the NF-κB pathway via RPS3 downregulation.** (**A**) Western blotting for NF-κB p65 in A2780 cells separately transfected with Vector, SIAH1, Clt-shRNA and sh-SIAH1 for 48 h. (**B**) Western blotting for NF-κB p65 in A2780 cells separately transfected with Vector, RPS3, Clt-shRNA and sh-RPS3 for 48 h. (**C**) Western blotting for SIAH1, RPS3 and NF-κB p65 in A2780 cells separately transfected with SIAH1and sh-SIAH1 for 48 h, and treated with PDTC (200 μM) for 24 h. A2780 cells were separately transfected with SIAH1 and sh-SIAH1 for 48 h and treated with PDTC for 24 h. The number of Cell Colonies was determined (**D**), the cell number of Transwell assay was obtained (**E**), the Wound closure percentage was calculated (0, 24, 48, 72 and 96 h) (**F**), and the Edu positive rates (**G**), cell viability (**H**, **I** left panels), and IC50 for cDDP (**H**, **I** right panels) were measured in A2780 cells. ^*^p < 0.05, ^**^p < 0.01, ^***^p < 0.001.

Considering that RPS3 can increase the transcriptional activity of NF-κB by binding its p65 subunit, we investigated whether SIAH1-mediated RPS3 degradation has an effect on the activity of NF-κB. Consistently, the results of obtained after RPS3 overexpression or knockdown revealed that the level of NF-κB protein exhibited an analogous trend with RPS3 in EOC cells (Figure 6A), whereas SIAH1 expression led to NF-κB downregulation, which was rescued by RPS3 overexpression (Figure 6B). Moreover, an inhibitor of NF-κB (PDTC) had no effect on SIAH1 and RPS3 expression (Figure 6C). All of these results indicate that SIAH1 inhibits the NF-κB pathway via RPS3 downregulation. Not surprisingly, PDTC enhanced the sensitization effect of SIAH1 and weakened the tumour-promoting effect of sh-SIAH1 (Figure 6D–6I). These findings suggest that the SIAH1-mediated degradation of RPS3 sensitizes EOC cells to cDDP partially by inhibiting the NF-κB pathway.

### SIAH1 enhances the sensitivity of EOC cells to cDDP by down-regulating RPS3 *in vivo*

**Figure 7:**
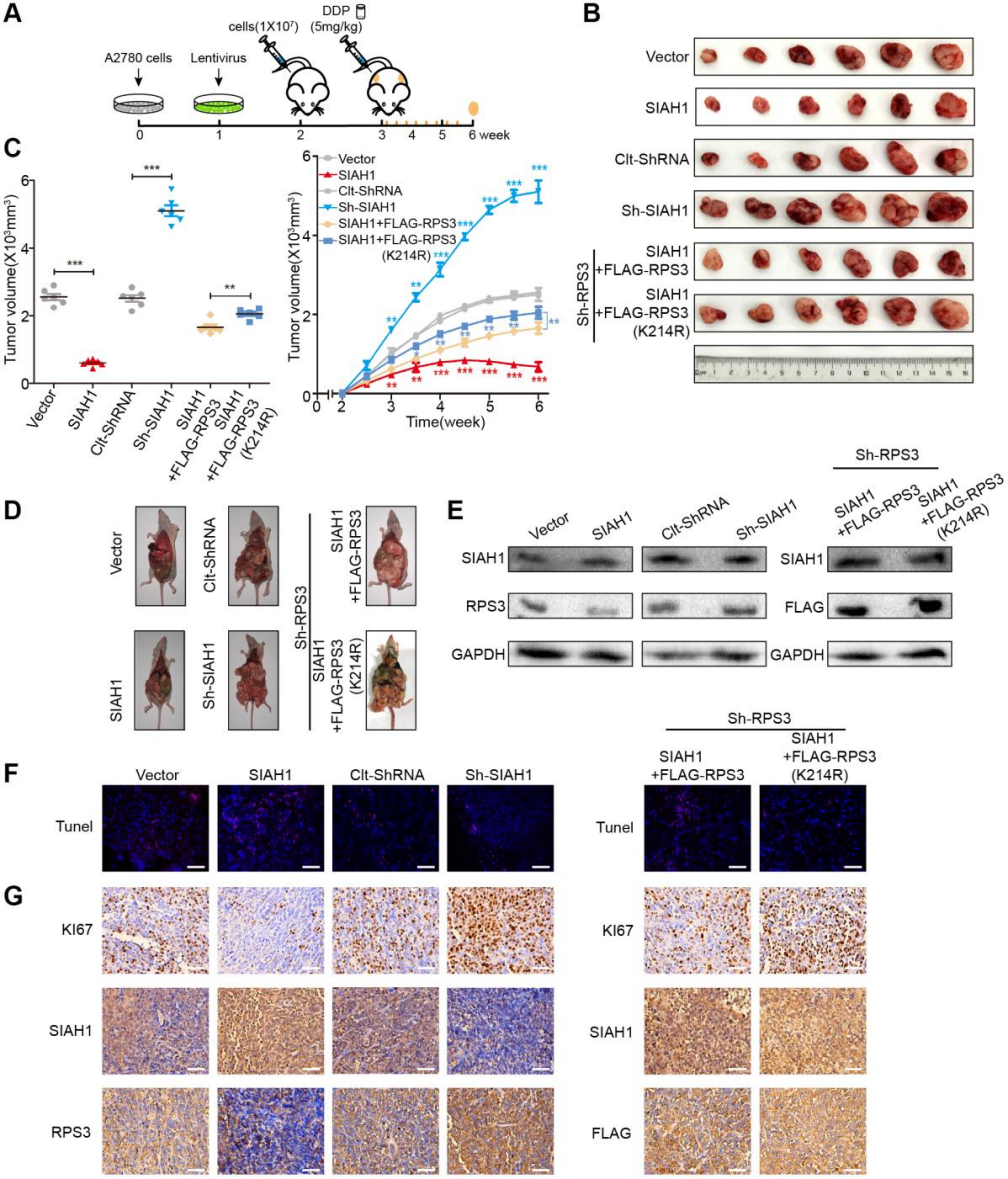
**SIAH1 enhances the sensitivity of ovarian cancer cells to cDDP by down-regulating RPS3 *in vivo*.** A2780 cells were transfected with Vector, SIAH1, Clt-shRNA, sh-SIAH1, SIAH1+FLAG-RPS3 and SIAH1+FLAG-RPS3 (K214R) lentivirus and were then subcutaneously and intraperitoneally injected into female BALB/c nude mice, and potential tumours were allowed to grow for one week (*n* = 6). All the groups were administered DDP (5 mg/kg) by intraperitoneal injection three times a week for a total of 8 times before sacrifice. (**A**) Flow chart of the animal experiment. (**B**) Representative images of the excised tumours on day 35 after tumour cell injection. (**C**) Tumour volumes of the excised tumours on day 35 (left) and the tumor growth curves (right) after tumour cell injection. (**D**) Typical pictures of tumours in the abdominal cavity after intraperitoneal injection in each group of mice. (**E**) The levels of SIAH1 and RPS3 proteins in mouse xenograft tumour tissues were assessed by Western blotting. (**F**) The apoptosis level of tumour tissue in each group was detected by TUNEL assay. Scale bar: 100 μm. (**G**) Immunohistochemistry analyses for KI67, SIAH1, RPS3 and FLAG staining were carried out with A2780 xenograft tumour sections. Representative staining is shown. Scale bar: 100 μm. ^**^p < 0.01, ^***^p < 0.001. Scale bar: 100 μm.

The above-described data jointly indicated that SIAH1 targeting represents an efficient anti-EOC therapy *in vitro*. To verify this hypothesis in a preclinical animal model, we developed an *in vivo* tumour model (Figure 7A) We subcutaneously and intraperitoneally injected A2780 cells transfected with Vector, SIAH1, Clt-shRNA or sh-SIAH1 lentivirus and A2780 cells in which RPS3 is stably knocked down transfected with SIAH1+FLAG-RPS3 or SIAH1+FLAG-RPS3 (K214R) lentivirus into female BALB/c nude mice with the aim of producing appropriately sized tumours (*n* = 6). Once tumours were formed (approximately one week), cDDP treatment was applied to these mice 3 times a week for a total of 8 times. These mice were then sacrificed, and the tumours were collected for analysis. Subsequently, we performed animal survival, tumour histology and immunostaining analyses.

SIAH1 overexpression resulted in a smaller tumours volume, fewer tumours, lower RPS3 protein expression, higher apoptosis rates and lower proliferation rates. The knockdown of SIAH1 exerted the opposite biological effect (Figure 7B–7G left panel). Compared with the SIAH1 + FLAG-RPS3 group, transfection with SIAH1 + FLAG-RPS3(K214R) resulted in a larger tumour volume, more tumours, higher RPS3 protein expression, lower apoptosis rates and higher proliferation rates (Figure 7B–7G right panel).

These results further support the hypothesis that SIAH1 enhances the sensitivity of EOC cells to cDDP by down-regulating RPS3 *in vivo*. Together, these *in vivo* results strongly favour the efficacy of SIAH1 overexpression combined with cDDP in anti-EOC therapy.

## DISCUSSION

**Figure 8:**
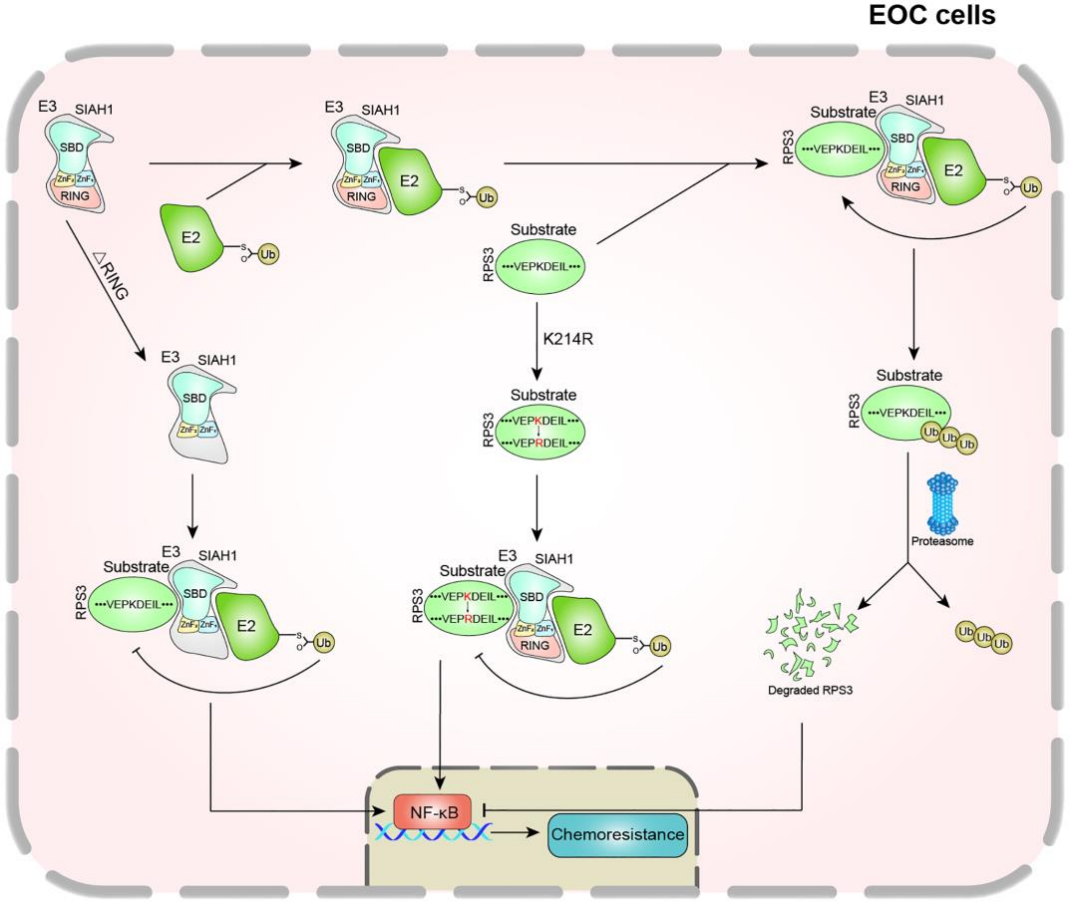
Schematic diagram of this study.

The development of acquired chemoresistance and enhanced metastasis is considered the major contributor to clinical mortality in EOC [2, 47]. As reported, cisplatin resistance in tumours is associated with increased drug efflux, altered intercellular signalling, tubulin mutation, and overexpression of the β-tubulin isotype composition [48, 49]. It has been reported that the ubiquitin-proteasome system (UPS) may be involved in the regulation of tumour occurrence and metastasis [50–52]. However, whether UPS increases the occurrence of EOC and cisplatin resistance is unclear. Here, we investigated the contributions and clinical relevance of SIAH1, which is an E3 ubiquitin ligase, in the context of chemoresistance. Here, we provide further information on the functional role of SIAH1, a significant conditioner of the UPS, in EOC resistance and provide conclusive evidence showing that SIAH1 is down-regulated in drug-resistant EOC cells, patient serum and cancer tissues. Low SIAH1 protein expression was found to be significantly related to the proliferation ability, invasiveness, migration and drug resistance of EOC cells. With regard to the mechanisms, RPS3 was identified as a new substrate of SIAH1 and was targeted by SIAH1 for ubiquitin proteasome-dependent degradation. Low RPS3 protein expression further inhibited the NF-κB pathway and led to the chemosensitization of EOC (Figure 8).

As a member of the highly conserved family of E3 ubiquitin ligases, an increasing number of studies have revealed the key role of SIAH1 in cancer development and tumour resistance. As documented, SIAH1 overexpression induced the apoptosis of cancer cells and inhibited the progression of cancer in many cancers, such as breast cancer and hepatocellular carcinoma [53, 54]. Our research confirmed that the exogenous overexpression of SIAH1 suppressed EOC cell proliferation, invasion, migration, drug resistance and tumour growth both *in vitro* and *in vivo*. In addition, compared with sensitive EOC patients, SIAH1 was significantly reduced in the serum and cancer tissues of drug-resistant EOC patients. Furthermore, SIAH1 overexpression clearly enhanced the effect of cDDP on apoptosis and tumour growth, whereas SIAH1 knockdown had the opposite effect.

RPS3 was identified as a potential SIAH1 binding protein by LC-MS/MS. Furthermore, our results showed that SIAH1 played an important biological role in the human body by inducing RPS3 ubiquitination and degradation. This study provides the first demonstration that the ribosomal protein RPS3 is an important substrate of SIAH1 and an important mediator through which SIAH1 regulates EOC cell proliferation, migration, invasion and drug resistance. As a ribosomal component, RPS3 plays a pivotal role in certain human tumours. YongJoong Kim et al., discovered that the level of secreted RPS3 is higher in more malignant cells and suggested that the secreted RPS3 protein is a poor prognostic indicator of malignant tumours [55]. Our results indicate that RPS3 may act as a tumour resistance promoter downstream of SIAH1 by promoting NF-κB-induced transcriptional activation, which is considered to contribute to EOC chemotherapy resistance. In addition, RPS3 overexpression could compensate for the reduction in EOC cell proliferation, migration, invasion and drug resistance caused by the overexpression of SIAH1, which indicates that SIAH1 and RPS3 jointly regulate the development and resistance of EOC. Further exploration of its mechanism revealed that SIAH1, which serves as a proverbial E3 ubiquitin ligase, induced RPS3 ubiquitination and degradation based on its RING domain. Consistent with this notion, the canonical E3 ubiquitin ligases, which contain the RING finger domain, induce the correlative substrate for ubiquitination and degradation via its conserved Cys and His residues [56].

Ubiquitin, which is attached to the lysine residue on a substrate, is recognized and degraded by the proteasome pathway [57, 58]. The specific lysine site of RPS3 for binding to ubiquitin was identified by LC-MS/MS and replaced by arginine. We concluded that lysine-214 of RPS3 is needed for SIAH1-induced ubiquitination and degradation. Animal tumour formation experiments, verified the effects of SIAH1 and RPS3 on tumour growth and development *in vivo*. RPS3 compensated for the inhibitory effect induced by SIAH1, whereas RPS3 (K214R) exerted a more obvious compensation effect than the former because it was not degraded by SIAH1. All of these experiments further focused on RPS3 at its lysine site K214. In the foreseeable future, lysine-214 of RPS3 may be a key site for predicting chemoresistance and prognosis of EOC based on SIAH1 deregulation.

## CONCLUSIONS

Collectively, these results revealed SIAH1 as an extraordinary and pivotal regulator of chemoresistance in EOC and stress the potential of the SIAH1-RPS3-NF-κB axis, which acts as a promising therapeutic target in response to chemotherapy in EOC patients, particularly those with lower SIAH1 expression.

## MATERIALS AND METHODS

### Cell culture and treatment

Human ovarian cancer cell lines (A2780 and SKOV3) and human renal epithelial cell lines (HEK-293T) were obtained from Shanghai Institute of Cell Biology, China Academy of Sciences. The cells were cultured in modified RPMI medium supplemented with 10% foetal calf serum (Gibco BRL, Grand Island, NY, USA), 100 U/mL penicillin and 100 μg/mL streptomycin in a humidified atmosphere containing 5% CO_2_ at 37°C.

### Plasmids and lentivirus

The GFP-SIAH1, FLAG-SIAH1, SIAH1, GFP-SIAH1 (∆RING) (knock out the RING domain), sh-SIAH1, FLAG-RPS3, Cherry-RPS3, FLAG-RPS3(K214R) (point mutant), and sh-RPS3 plasmids and their corresponding negative control plasmids were purchased from Genechem Co., Ltd. (Shanghai, China). Lentiviruses for SIAH1, sh-SIAH1, RPS3 and RPS3 (K214R) and their corresponding negative control lentiviruses were purchased from Genechem Co., Ltd. (Shanghai, China).

### Tissue samples

With the approval of the Jiangsu University ethics committee, serous ovarian cancer samples from patients with FIGO stage III or IV were collected at Zhenjiang Maternal and Child Health Hospital (The Fourth Affiliated Hospital of Jiangsu University) and The Affiliated People's Hospital of Jiangsu University. All the patients were administered the standard platinum-based therapy after surgery, and informed consent was obtained from all the patients. The PFS was calculated from the time of surgery to the time of progression or recurrence. Platinum resistance or platinum sensitivity was defined as a relapse or progression within 6 months or 6 months after the last platinum-based chemotherapy, respectively. Each group had more than 12 patient samples. Clinical and pathological features are described in Supplementary Table 5.

### Transfection

The details are described in the Supplementary Methods.

### Colony forming assay

The details are described in the Supplementary Methods.

### Wound-healing assay

The details are described in the Supplementary Methods.

### Assessment of chemosensitivity to cDDP

The details are described in the Supplementary Methods.

### Evaluation of apoptosis

The details are described in the Supplementary Methods.

### Cycloheximide (CHX) chase assay

The details are described in the Supplementary Methods.

### Immunohistochemistry and scoring

The details are described in the Supplementary Methods.

### Western blotting analysis

The details are described in the Supplementary Methods.

### Real-time Quantitative PCR

The details are described in the Supplementary Methods.

### LC-MS/MS analysis

In-gel digestion.LC-MS/MS analysis was performed by Shanghai Genechem Co., Ltd. The gel was cut into 48 slices from which proteins were digested, and the resulting peptides were extracted and lyophilized before further analysis. Peptide powders were resuspended in solvent A (2% acetonitrile and 0.1% formic acid in water) and loaded onto a C18 reversed-phase column (100 μm in diameter, 15 cm long, 3 μm resin from Michrom Bioresources, Auburn, CA, USA). Each peptide mixture was separated with a linear gradient of solvent B (5–15%) for 15 min, a gradient from 15–35% for 85 min, and 90% for 20 min. The eluted peptides were injected directly into an LTQ-Orbitrap XL (Thermo Fisher Scientific, Inc.) through a nano-electrospray ion source (Proxeon Biosystems) with a voltage of 1.85 kV and a transfer capillary temperature of 200°C. Data were acquired using X calibur software (Thermo Electron) in the data-dependent mode. An accumulation of 106 ions was needed to trigger a full MS scan, with a maximum accumulation time of 500 ms and a resolution of 60,000 (m/z 400), ranging from 400–2,000 Da. The six most intensive ions per MS scan were selected and fragmented by CID in LTQ to perform the MSMS scan with an accumulation of at least 5,000 ions and a maximum accumulation time of 100 ms. The normalized collision energy was 35%, activation Q was 0.25, activation time was 30 ms, and dynamic exclusion was enabled with a maximum retention period of 90 s and a relative mass window of 10 ppm. A lock mass (PCM, MW445.12) was introduced to improve the mass accuracy of survey scans.

### *In vitro* ubiquitination and co-immunoprecipitation

For *in vitro* ubiquitination, HEK293T cells were transfected with plasmids as described previously for 36 h. The cells were then treated with 15 μM MG132 (MedChemExpress, Monmouth Junction, NJ, USA) for 6 h and lysed in buffer on ice for 30 min with oscillation for 30 s at 5 min intervals. The samples were centrifuged for 10 min at 12,000 g at 4°C, and the supernatants were collected. A total of 0.5 mg of lysate was incubated overnight at 4°C with PureProteome™ Protein A/G Magnetic Beads (Millipore, Bedford, MA, USA) and antibodies against SIAH1 (Abcam, Cambridge, UK), RPS3 (Abcam, Cambridge, UK), GFP (Proteintech, Rosemont, IL, USA) or FLAG (Cell Signaling Technology, Danvers, MA, USA). After six washes with lysis buffer, the immuneprecipitates were resuspended in 5× loading buffer, degenerated and further analysed by Western blotting of cell lysates. Immunoblotting was performed using ubiquitin, SIAH1, RPS3, GFP, FLAG, and GAPDH antibodies.

### Experimental animals

Female specific pathogen-free BALB/c nude mice weighing 150 g were provided by the Shanghai SLAC Experimental Animal Co., Ltd. The nude mice were housed at room temperature (26 ± 2°C) with a humidity of 45–55% and a light duration of 12 h in the laboratory animal centre of Jiangsu University. All experimental procedures involving nude mice were approved by the Institutional Animal Care and Use Committee of Jiangsu University.

### Statistical analysis

All numeric data are presented as the means ± standard deviations (SDs) of at least three independent experiments. The experimental results were analysed by analysis of variance or two-tailed Student’s *t* test at a significance level of *P* < 0.05 (^*^P < 0.05, ^**^P < 0.01 and ^***^P < 0.001). Prism 5 software (GraphPad Software, San Diego, CA, USA) was used to perform all the statistical analyses. A *P* value < 0.05 was considered to indicate statistical significance.

### Consent for publication

All authors reviewed the manuscript and consented publication in this journal.

### Data availability

The raw data supporting the results of this study will be made available by the authors without undue reservation.

## Supplementary Materials

Supplementary Methods

Supplementary Figures

Supplementary Tables 3-5
