# Supplementary material for: SIAH1-mediated RPS3 ubiquitination contributes to chemosensitivity in epithelial ovarian cancer
Source: Aging (Albany NY). 2022 Aug 8;14(15):6202–26. doi: 10.18632/aging.204211 (PMC9417229; doi:10.18632/aging.204211)
Supplement: Supplementary Methods [file aging-14-204211-s001.pdf]

SUPPLEMENTARY METHODS

Transfection

Cells were seeded into 6-well plates (6 × 10^5 cells/well) and transfected with GFP-SIAH1, FLAG-SIAH1, Sh-SIAH1, Cherry-RPS3, FLAG-RPS3, FLAG-RPS3 (K214R), Sh-RPS3 plasmids and their negative controls (all plasmids from Gene Pharma Company, Shanghai, China) at a final concentration of 100 nM using Lipofectamine 2000 (Invitrogen, Carlsbad, CA, USA). The details on plasmids are described in Supplementary Table 1.

Colony forming assay

The cells were plated in 6-well dishes (500 cells/well) and exposed to a specific dose of cDDP(0.5 μg/ml), and subsequently grown for 14 days. Next, the cells were fixed with 4% Paraformaldehyde (PFA), which were then stained with 0.3% crystal violet. Colonies containing more than 50 cells were identified using densitometry software and scored as survivors.

Wound-healing assay

To evaluate cell motility, 6 × 10^5 cells were seeded into 6-well plates and serum-starved for 24 h at full confluency. A sterile 200 ml pipette tip was used to scratch the cells to form a wound. The cells were washed with PBS and cultured in serum-free medium. Images were captured at regular intervals to evaluate wound closure. The images were evaluated using Image J software to measure the wound area at various time points. The percentage of wound closure was calculated as the wound area at a given time compared with the initial wound surface. Images shown are representative of three independent experiments performed in duplicate.

Assessment of chemosensitivity to cDDP

Cells were plated into 96-well plates (5 × 10^3 cells/well) and exposed to various doses of cDDP (1, 2, 4, 8, 16 and 32 μg/ml). Then, 10 μl of CCK-8 solution (Vazyme, Nanjing, China) was added to each well, and the plate was incubated for 2 h in a humidified incubator. The absorbance of each well was measured at 450 nm using a Model 550 series microplate reader (Bio-Rad Laboratories, USA). Cell viability was expressed as the ratio of treated cells to untreated controls at each dose or concentration. The IC50 value for each cell line was determined by nonlinear regression analysis using GraphPad Prism (GraphPad Software Inc., San Diego, CA, USA).

Evaluation of apoptosis

To evaluate cell motility, 6 × 10^5 cells were seeded into 6-well plates and analysed by the EDU (5-Ethynyl-2′-deoxyuridine) cell proliferation test kit (Ribobio, Guangzhou, China).

Cycloheximide (CHX) chase assay

Cells transfected with plasmids for 36 h. 40 mM CHX was then added to each well. Whole cell lysates were prepared at various time points after CHX treatment and subjected to western blotting assay with indicated antibodies.

Immunohistochemistry and scoring

The tumors were formalin fixed, embedded in paraffin, and sectioned and then heat-immobilized or pepsin-immobilized according to the manufacturer's instructions. The slides were stained with hematoxylin (H&E) or incubated with antibodies against SIAH1 (Abcam, Cambridge, UK), RPS3 (Abcam, Cambridge, UK) or Ki67 (Santa Cruz Biotechnology, Santa Cruz, CA, USA) and then detected using the Dako Envision two-step method of immunohistochemistry (Carpinteria, CA, USA). We divided the positive staining results into 0–4 categories as following: 0: <5%; 1: 6–25%; 2: 26–50%; 3: 51–75%; and 4: >76% staining.

Western blotting analysis

Proteins from cells, Ovarian cancer tissues, Serum samples and xenograft tumors were performed in 100 ul RIPA lysis buffer (high) (Solarbio, Beijing, China) which added with 1ul Proteasome inhibitor (Solarbio, Beijing, China). Proteins were separated by sodium dodecyl sulfate-polyacrylamide gel electrophoresis and transferred onto polyvinylidene difluoride membranes (Bio-Rad Laboratories, USA). After blocking, the membranes were immunoblotted with SIAH1 (Abcam, Cambridge, UK), RPS3 (Abcam, Cambridge, UK), P65 (Wanleibio, Shanghai, China), P50 (Wanleibio, Shanghai, China), GFP (Proteintech, Rosemont, IL, USA) or FLAG (Cell Signaling Technology, Danvers, MA, USA) primary antibodies and then with horseradish peroxidase-conjugated secondary antibodies. GAPDH (Proteintech, Rosemont, IL, USA) was used as an endogenous control. The Odyssey Infrared Imaging System was used to visualize the targeted protein bands.

Supplementary Table 1. Details on plasmids.

Plasmids	Source	Species	Transcripts	Element sequence
GFP-SIAH1	Gene Pharma Company	Human	NM_001006610	CMV-MCS-3FLAG-IRES-EGFP-SV40-Neomycin
FLAG-SIAH1	Gene Pharma Company	Human	NM_001006610	CMV-MCS-3FLAG-SV40-Neomycin
GFP-SIAH1 (ΔRING)	Gene Pharma Company	Human	NM_003031 (del41-76aa)	CMV-MCS-polyA-EF1A-zsGreen-sv40-puromycin
Sh-SIAH1	Gene Pharma Company	Human	NM_001006691-1	hU6-MCS-CMV-GFP-SV40-Neomycin
Cherry-RPS3	Gene Pharma Company	Human	NM_001005	CMV-MCS-IRES-Cherry-SV40-NeomycinNM_001006610
FLAG-RPS3	Gene Pharma Company	Human	NM_001005	CMV-MCS-3FLAG-SV40-Neomycin
FLAG-RPS3 (K214R)	Gene Pharma Company	Human	NM_001005 (K214)	CMV-MCS-3FLAG-polyA-EF1A-Cherry-SV40-Puromycin
Sh-RPS3	Gene Pharma Company	Human	NM_001260507	U6-MCS-Ubiquitin-Cherry-IRES-puromycin

Supplementary Table 2. Primers used for qRT-PCR.

PRIMER	FORWARD (5′–3′)	REVERSE (5′–3′)
RPS3	GCATCTTCAAAGCTGAACTGAA	CTTCTGAACTACAGCAGTCAG

Real-time Quantitative PCR

Total RNA was isolated using Trizol reagent. cDNA as synthesized using a FastQuant RT Kit (with gDNase) (#KR106, Tiangen, Shanghai, China) according to the manufacturer's instructions. Quantitation of mRNAs was carried out using a miRcute Plus mRNA qPCR Detection Kit (#FP411, Tiangen). The raw qRT-PCR mRNAs data were normalized to the spiked GAPDH levels as described previously. The quantitative PCR procedures were carried out with real-time PCR SYBR Green q-PCR Super-mix. The mRNA expression levels were analyzed and quantified by calculating using the $2^{-\Delta\Delta}$Ct method. The primers of mRNA are listed in Supplementary Table 2.
