# Supplementary material for: SIAH1-mediated RPS3 ubiquitination contributes to chemosensitivity in epithelial ovarian cancer
Source: Aging (Albany NY). 2022 Aug 8;14(15):6202–26. doi: 10.18632/aging.204211 (PMC9417229; doi:10.18632/aging.204211)
Supplement: Supplementary Figures [file aging-14-204211-s002.pdf]

SUPPLEMENTARY FIGURES

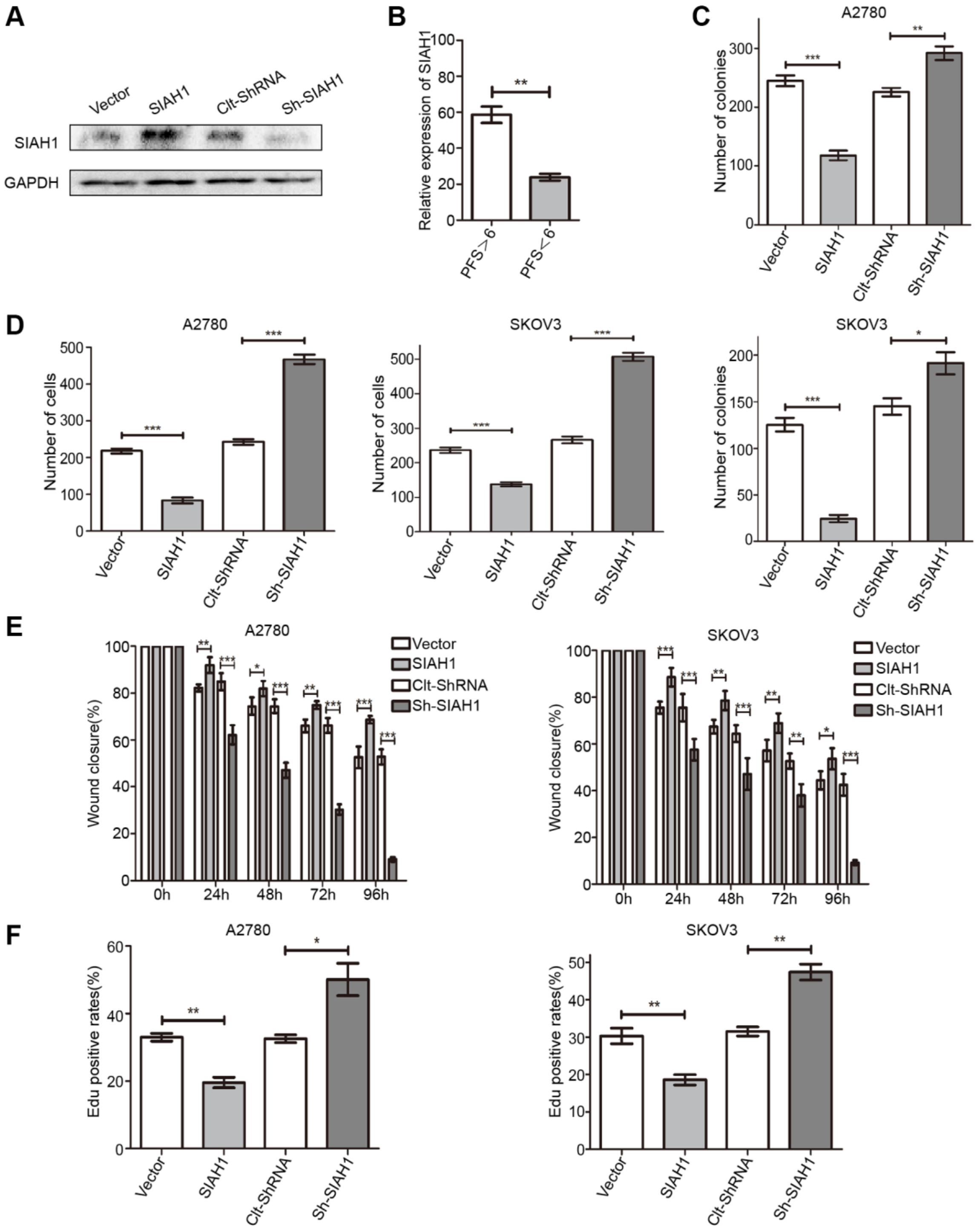

Supplementary Figure 1. SIAH1 sensitizes ovarian cancer cells to cDDP. (**A**) Western blotting for SIAH1 in A2780 cells transfected with Vector, SIAH1, Clt-shRNA and sh-SIAH1 were shown. (**B**) Relative SIAH1 expression in tumor specimens form ovarian cancer patients with PFS > 6 months vs. PFS < 6 months. A2780 cells and SKOV3 cells were separately transfected with Vector, SIAH1, Clt-shRNA and sh-SIAH1 for 48 h, the number of cell colons (**C**), the cell number of transwell (**D**), the calculation of wound closure percentage (0, 24, 48, 72 and 96 h) (**E**), Edu positive rates (**F**) were measured in A2780 cells and SKOV3 cells respectively. $^{*}p < 0.05$, $^{**}p < 0.01$, $^{***}p < 0.001$.

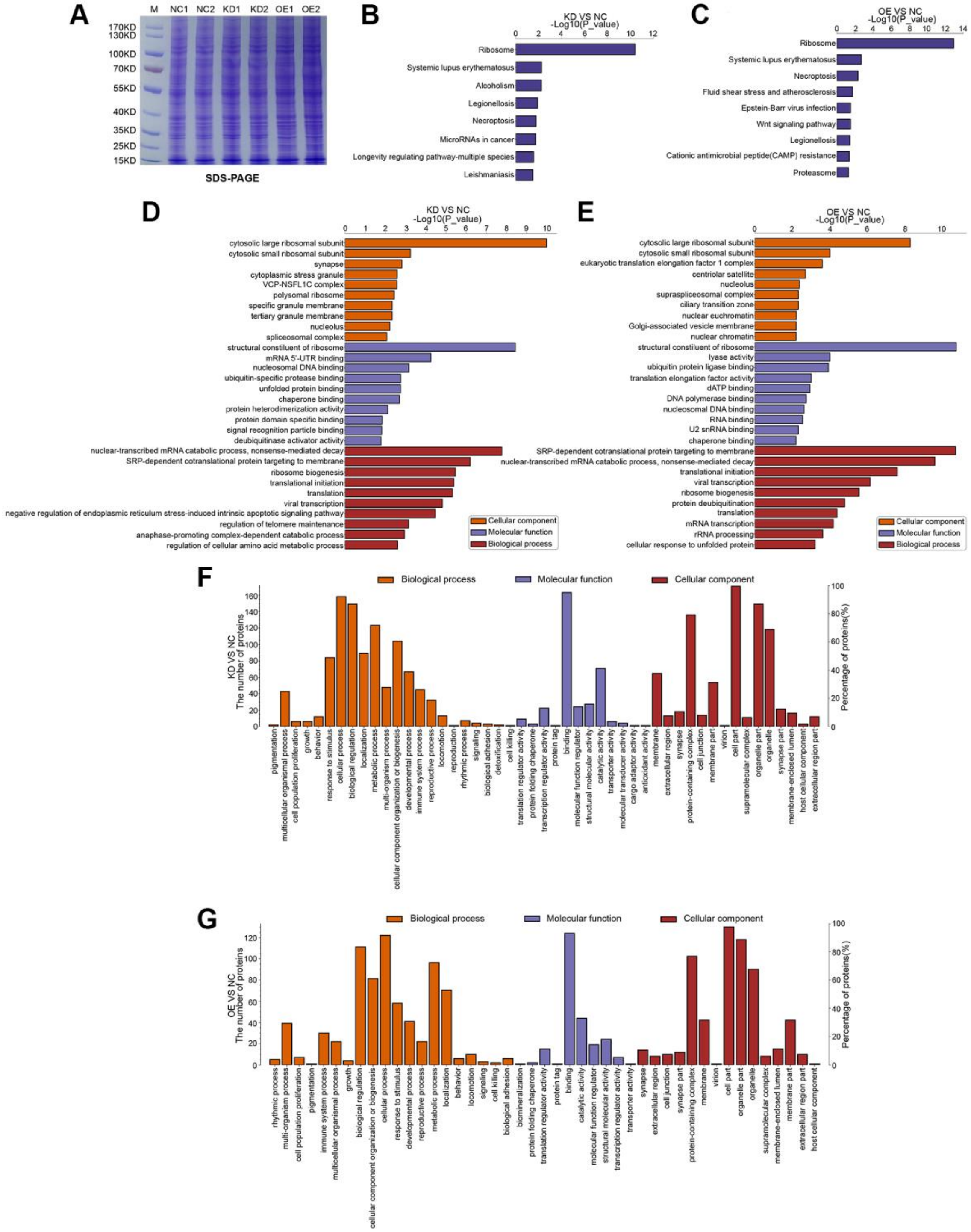

Supplementary Figure 2. Advanced bioinformatics analysis with SIAH1 knockdown or overexpression. (**A**) SDS-PAGE assay was performed for the six samples (NC1, NC2, KD1, KD2, OE1 and OE2) in HEK-293T cells. (**B**) Significantly enriched KEGG pathway statistics (KD vs. NC). (**C**) Significantly enriched KEGG pathway statistics (OE vs. NC). (**D**) Significantly enriched GO term statistics (KD vs. NC) (top 10). (**E**) Significantly enriched GO term statistics (OE vs. NC) (top 10). (**F**) Level 2 Statistics of GO annotation results (KD vs. NC). (**G**) Level 2 Statistics of GO annotation results (OE vs. NC).

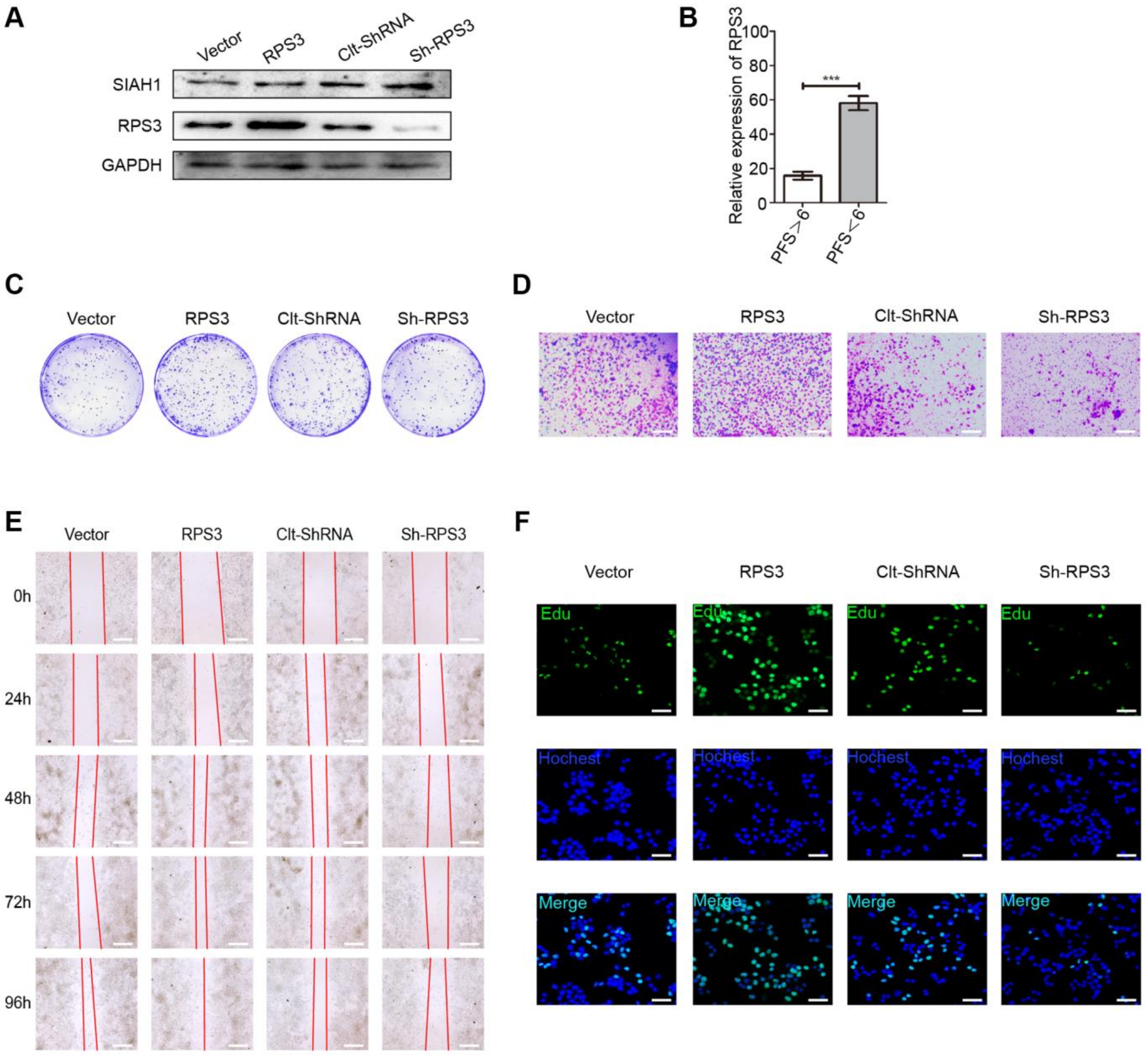

Supplementary Figure 3. SIAH1 down-regulates the protein level of RPS3. (**A**) Western blotting for SIAH1 and RPS3 in A2780 cells transfected with Vector, RPS3, Clt-shRNA and sh-RPS3 for 48 h. (**B**) Relative RPS3 expression in tumor specimens form ovarian cancer patients with PFS > 6 months vs. PFS < 6 months. Cell colonies assay (**C**) and Transwell assay (**D**) of A2780 cells transfected with Vector, RPS3, Clt-shRNA and sh-RPS3 were shown. Scale bar: 400 µm. The wound-healing assay was used to assess the effects of RPS3 on cellular motility over time as shown (0, 24, 48, 72 and 96 h; Scale bar: 400 µm) (**E**) and Cell Edu assay (Scale bar: 200 µm) (**F**) were analyzed in A2780 cells respectively. $^{***}p < 0.001$.
