# Supplementary material for: SIAH1-mediated RPS3 ubiquitination contributes to chemosensitivity in epithelial ovarian cancer
Source: Aging (Albany NY). 2022 Aug 8;14(15):6202–26. doi: 10.18632/aging.204211 (PMC9417229; doi:10.18632/aging.204211)
Supplement: Supplementary Tables 3-5 [file aging-14-204211-s003.pdf]

SUPPLEMENTARY TABLES

Supplementary Table 3. The potential SIAH1-interacting proteins.

Gene Name	Unique peptides	Sequence coverage [%]
RPS3	25	75.7
HNRNPA2B1	27	76.8
RPL7	23	70.2
HIST2H2AB	2	70
NME2	6	75
PFN1	13	78.6
NPM1	24	75.5
ENO1	27	71.2
RPLP2	8	80
NEDD8	7	81.5
HIST3H2BB	2	76.2
PRDX1	12	79.9
RPL24	22	73.2
UBA52	2	71.1
RPS27A	7	73.1
PPIA	17	85.5
HIST1H4A	16	72.8
UBE2N	12	70.4
VIM	36	72.3
PRDX6	17	72.3

Supplementary Table 4. The potential ubiquitination sites.

Gene Name	Positions within proteins	Score	GlyGly (K) Probabilities	OE/NC	*P* value (Significance A)
RPS3	214	176.53	KPLPDHVSIVEPK(1)DEILPTTPISEQK	3.21965	0.00983847
RPS3	10	106.68	K(1)FVADGIFK	0.33357	0.0373192
RPS3	8	91.626	AVQISK(1)K(1)R	0.315526	0.0281246
RPS3	141	146.79	GCEVVVSGK(1)LR	0.284557	0.0161287
RPS3	230	133.32	GGK(1)PEPPAMPQPVPTA	0.916819	0.981948
RPS3	202	63.894	K(1)PLPDHVSIVEPK	0.642557	0.45523
RPS3	90	70.96	FGFPEGSVELYAEK(1)VATR	0.593273	0.363198
RPS3	62	147.2	TQNVLGEK(1)GRR	0.495039	0.201236
RPS3	197	100.55	IMLPWDPTGK(0.992)IGPK(0.008)	0.432506	0.120391
RPS3	75	222.08	IRELTAVVQK(1)R	0.385161	0.0735663
RPS3	7	97.068	AVQISK(1)K(1)R	0.359152	0.0533932

Supplementary Table 5. Clinical and pathological features of EOC patients.

Characteristics	Age (years)	Pathological type	FIGO stage	Grade	Progression-free survival (months)
EOC1	66	serous mucinous carcinoma	IIIA1(i)	Low	>6
EOC2	62	serous carcinoma	IIIA2	Moderate	>6
EOC3	70	mucinous carcinoma	IVA	High	>6
EOC4	65	serous carcinoma	IIIC	High	>6
EOC5	63	serous mucinous carcinoma	IIIC	Moderate	>6
EOC6	64	serous carcinoma	IVA	Moderate	>6
EOC7	68	serous carcinoma	IIIC	High	>6
EOC8	67	clear cell carcinoma	IVA	High	>6
EOC9	69	endometrioid carcinoma	IIIA1(ii)	Low	>6
EOC10	71	mucinous carcinoma	IIIC	Moderate	>6
EOC11	65	serous carcinoma	IVA	Low	>6
EOC12	59	serous carcinoma	IVA	High	>6
EOC13	60	serous carcinoma	IIIC	High	<6
EOC14	68	serous mucinous carcinoma	IIIC	Low	<6
EOC15	68	mucinous carcinoma	IVA	High	<6
EOC16	65	clear cell carcinoma	IIIC	High	<6
EOC17	66	serous carcinoma	IIIB	Moderate	<6
EOC18	69	serous carcinoma	IIIA2	Moderate	<6
EOC19	72	serous carcinoma	IIIC	High	<6
EOC20	66	serous carcinoma	IIIB	High	<6
EOC21	70	serous carcinoma	IVB	High	<6
EOC22	65	serous carcinoma	IIIC	Moderate	<6
EOC23	76	serous carcinoma	IIIC	High	<6
EOC24	60	serous carcinoma	IVA	High	<6
